# The Carbon Footprint of Conference Papers

**DOI:** 10.1371/journal.pone.0066508

**Published:** 2013-06-26

**Authors:** Diomidis Spinellis, Panos Louridas

**Affiliations:** 1 Department of Management Science and Technology, Athens University of Economics and Business, Athens, Greece; 2 Greek Research and Technology Network, Athens, Greece; The Ohio State University, United States of America

## Abstract

The action required to stem the environmental and social implications of climate change depends crucially on how humankind shapes technology, economy, lifestyle and policy. With transport CO_2_ emissions accounting for about a quarter of the total, we examine the contribution of CO_2_ output by scientific travel. Thankfully for the reputation of the scientific community, CO_2_ emissions associated with the trips required to present a paper at a scientific conference account for just 0.003% of the yearly total. However, with CO_2_ emissions for a single conference trip amounting to 7% of an average individual’s total CO_2_ emissions, scientists should lead by example by demonstrating leadership in addressing the issue.

## Introduction

The environmental and social implications of climate change depend not only on Earth’s systemic responses, but also on how humankind shapes technology, economy, lifestyle and policy [1]. Action should not be postponed, as it is argued that we have already surpassed a safe threshold in atmospheric carbon dioxide concentration (from a 280 ppm pre-industrial value to 387 ppm today, with a proposed boundary threshold of 350 ppm) [2]. Changes in economy, lifestyle, and policy, entail changes in human behaviour, which will ultimately require decisions involving moral questions. Decisions should not be put off, considering that decisions that delay mitigation may have the greatest effect on the cost-risk distribution for returning global temperature increase to sustainable levels [3]. Science has an important role in framing the discussion and informing policy makers and the public [4]. This work adds to the discussion by highlighting the contribution of science itself to global carbon dioxide output [5]; in particular, to investigate the annual contribution of CO_2_ output by travelling to scientific conferences to present a paper. These emissions could directly affect the environment, but also reflect badly on science, as demonstrated by the derisive press coverage of the 2009 Copenhagen summit’s CO_2_ footprint [6], [7].

In terms of policy, choices to mitigate climate change may focus on market mechanisms (e.g., subsidies, trading schemes, or taxes), information disclosure (e.g., energy efficiency labeling schemes), and behavioral science [8]. Our focus is on information disclosure. We examine emissions associated with scientists travelling to present their work at conferences that publish their proceedings through indexed imprints. This is a subset of their total travel, as a part of their travel miles involve non-conference travel. However, conference travel is integral to scientists’ work and, in contrast to other kinds of travel, its purpose is tied to science’s core function. Conference trips are also, at least in theory, discretionary in the sense that they can be substituted through the use of various communication technologies. Furthermore, the emissions we study are also a subset of the total travel associated with conferences, because some conferences do not publish indexed proceedings, and many scientists attend conferences without presenting a published paper. Extrapolating total conference travel from our data through the use of conference attendance figures is difficult, because, according to our experience, attendance at conferences by scientists who do not have a paper to present tends to be biased toward those living relatively near the conference’s location.

We show that CO_2_ emissions associated with the trips required to present papers at scientific conferences account for 0.003% of the yearly total travel emissions. This is a bit more than the total transportation emissions for Geneva in a recent year, at about 800 kt CO_2_ (1 kt is 10^6^ kg), or less than the total transportation emissions for Barcelona, at about 1236 kt CO_2_ [9]. Thankfully for the reputation of the scientific community, the environmental impact of the scientific conference trips we examine seems to be overblown. However, with CO_2_ emissions for a single conference trip amounting to 7% of an average individual’s total CO_2_ emissions, scientists should lead by example in addressing the issue.

## Results

We examine emissions associated with scientists travelling to present their work at conferences. We base our study on author and conference location data obtained from conference papers. We obtained our primary set of conference paper bibliographic details from the Scopus digital library by retrieving details of randomly sampled conference proceedings papers published over the period 1998–2008. This selection yielded a sample of 2.8% of the population’s papers.

**Figure 1:**
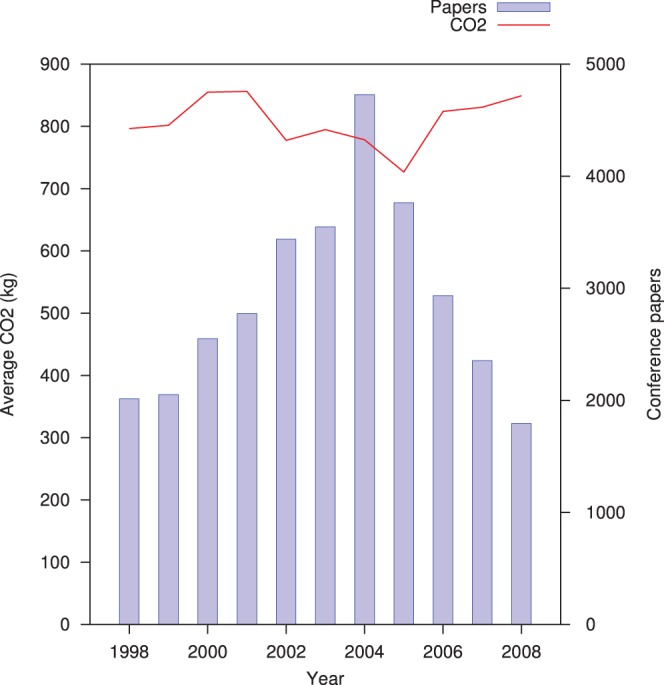
Conference travel CO_2_ per year.

In general, total air passengers per year increased dramatically from 2001 to 2008, with a negligible decline in 2002 and 2009 [10], [11] (Table 1). Over the same period, although average CO_2_ emissions of scientific conference travel fell from 2001 to 2005, they increased again to the year 2000 levels in 2008 (Figure 1 and Table 2 ). Over the year, although average emissions per paper are fluctuating, CO_2_ total emissions per month are considerably higher during the spring and autumn months, which are popular for holding conferences (Table 3).

**Table 1:** Air Passengers per Year.

Year	Passenger numbers, millions
2001	1640
2002	1639
2003	1776
2004	1982
2005	2123
2006	2233
2007	2418
2008	2485
2009	2479
2010	2681
2011	2830
2012F	2973
2013F	3128

**Table 2:** Geolocated Papers per Year and Corresponding Conference Travel CO_2_.

Year	Geolocated papers	Total papers	Geolocation %	Average CO_2_ kg
1998	2,014	4,110	49.0	796
1999	2,051	3,835	53.5	802
2000	2,550	4,620	55.2	855
2001	2,774	4,883	56.8	856
2002	3,437	5,482	62.7	778
2003	3,547	5,768	61.5	795
2004	4,729	7,724	61.2	778
2005	3,763	7,126	52.8	727
2006	2,934	6,338	46.3	824
2007	2,353	5,570	42.2	831
2008	1,793	4,504	39.8	849

**Table 3:** Conference Travel CO_2_ Output per Month.

Month	Papers	Average CO_2_ kg	Total CO_2_ kg
January	1,763	908	1,600,565
February	1,228	861	1,057,736
March	1,730	793	1,372,473
April	2,401	770	1,848,222
May	4,078	890	3,629,469
June	4,597	757	3,480,306
July	2,783	918	2,556,144
August	2,376	782	1,857,333
September	3,574	685	2,449,029
October	3,713	797	2,960,106
November	2,591	713	1,846,634
December	1,262	838	1,057,254

**Figure 2:**
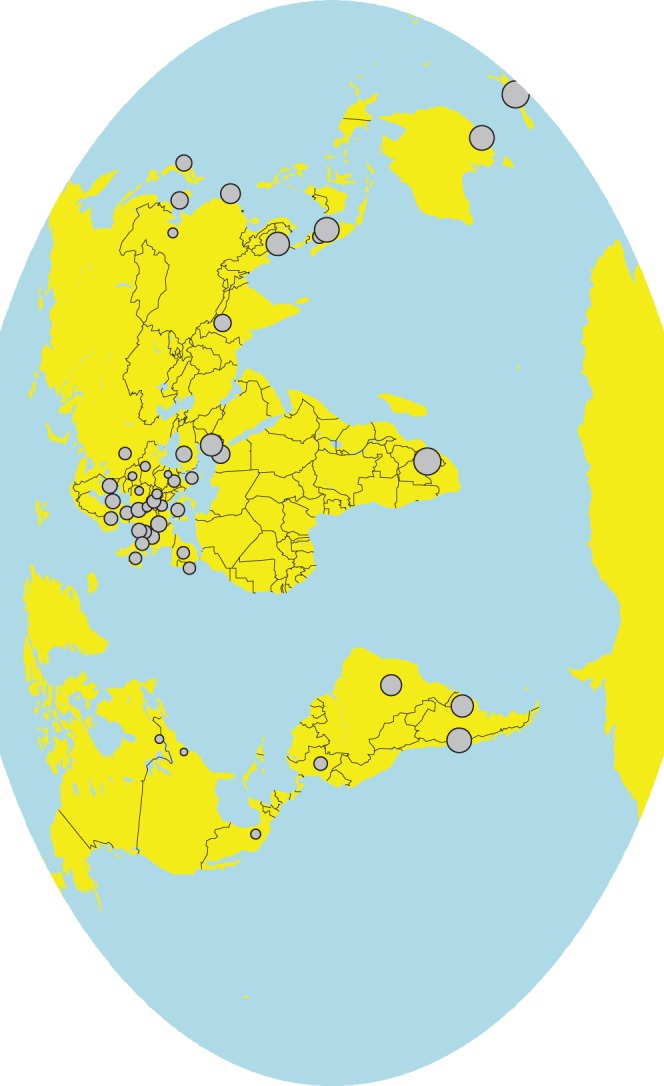
Average CO_2_ emissions for a paper to be presented by an author originating from a particular country.

Author countries in the southern hemisphere fare quite badly in terms of the associated CO_2_ emissions, while author countries with low emissions are those near conference locations (us, Canada, Mexico); see Figure 2, Table 4 and Table 5. However, we found no correlation between country wealth [12] and average emissions per paper–a country may lack financial resources, but when its scientists travel they do not necessarily fly less miles. At the same time, the papers published by authors in the country are correlated with country wealth in logarithmic transformation (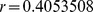, 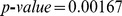, 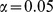, 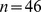, Pearson correlation test, permutation test used for hypothesis testing, 100,000 sampled permutations) and are therefore also correlated with the total emissions due to papers published by authors in the country (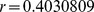, 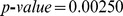, 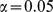, 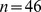).

**Table 4:** Worst Average CO_2_ Emissions by Author Country.

Country	Average CO_2_ kg	# Samples
South Africa	1,891	30
New Zealand	1,880	51
Australia	1,722	312
Chile	1,711	61
Singapore	1,669	491
Thailand	1,580	41
Argentina	1,535	33
Israel	1,483	210
Brazil	1,403	151
Taiwan	1,369	145

**Table 5:** Best Average CO_2_ Emissions by Author Country.

Country	GDP per capita $ PPP	Avg CO_2_ kg	# Samples
Estonia	17,695	479	28
United States	45,934	510	12,127
Romania	11,869	515	67
Belarus	12,750	592	30
Poland	18,050	622	258
Canada	37,947	622	1,313
China	6,778	668	2,315
Hungary	18,506	668	89
Czech Republic	24,271	689	94
Mexico	13,609	716	22

Two factors seem to increase the CO_2_ emissions associated with a conference location: distance and popularity (Table 6); at the country level (Table 6) the southern hemisphere again fares particularly badly. On the other hand, conference countries and locations (Table 7) associated with low CO_2_ emissions are those located off the beaten track.

**Table 6:** Worst Average CO_2_ Emissions by Conference Country and Location.

Country			Location			
	Average	#			Average	#
Name	CO_2_ kg	Samples	City	Country	CO_2_ kg	Samples
Australia	1,902	461	Sydney	AS	2,010	307
Argentina	1,795	62	Adelaide	AS	1,827	42
Brazil	1,403	77	San Juan	AR	1,813	52
Thailand	1,137	87	Melbourne	AS	1,766	63
Taiwan	1,081	128	Hyderabad	IN	1,550	22
Mexico	1,041	168	Rio de Janeiro	BR	1,516	45
Turkey	912	179	Vancouver	US	1,327	44
Switzerland	907	112	Honolulu	US	1,290	645
India	876	127	Marina del Rey	US	1,265	23
United States	875	19,350	Rochester	US	1,255	59

**Table 7:** Best Average CO_2_ Emissions by Conference Country and Conference Location.

Country			Location			
	Average	#			Average	#
Name	CO_2_ kg	Samples	City	Country	CO_2_ kg	Samples
Serbia	180	29	Kumamoto	JA	48	25
Croatia	278	38	Toyama	JA	68	60
Ukraine	312	27	Yamagata	JA	83	73
Russia	344	198	Bled	SI	167	21
Poland	349	196	Wuhan	CH	218	186
China	391	1,925	Dalian	CH	218	72
Slovenia	445	51	Hefei	CH	241	33
Romania	480	69	Aveiro	PO	247	26
Hungary	511	89	Dresden	GM	248	43
Ireland	518	74	Jinan	CH	249	46

A location’s popularity as a conference location (Table 8) doesn’t seem to be associated with travel distance and the consequent CO_2_ emissions (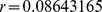, 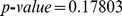, 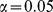, 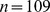, test between the average CO_2_ emissions of a location and number of papers presented there, Pearson correlation test, permutation test used for hypothesis testing, 100,000 sampled permutations). None of the low CO_2_ locations appear in the list of the ten most popular locations, while Honolulu, which is the eighth worst destination from a CO_2_ emission perspective, is also famously popular.

**Table 8:** Conference Travel CO_2_ Emissions of the Most Popular Locations.

City	Country	# Samples	Average CO_2_ kg
San Diego	US	1,828	972
San Francisco	US	1,364	955
San Jose	US	1,357	982
Boston	US	1,238	816
Orlando	US	1,170	779
Honolulu	US	645	1,290
Beijing	CH	644	420
Washington	US	542	793
Baltimore	US	497	767
Chicago	US	480	700

**Figure 3:**
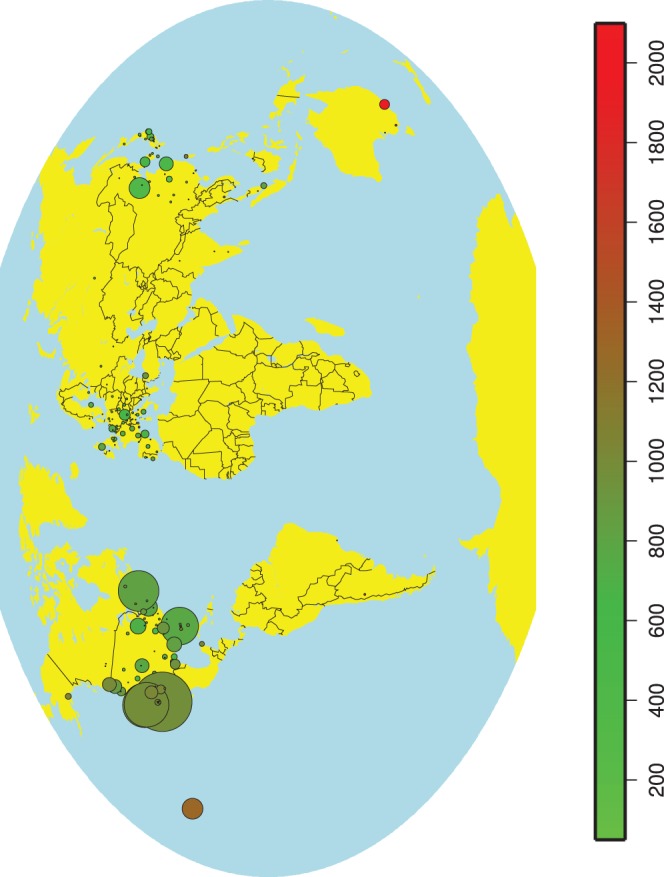
Average CO_2_ emissions for a paper to be presented at a conference location. The circle’s color represents the average CO_2_ emissions (kg), while the circle’s area is proportional to the number of papers presented at the particular location.

Although the CO_2_ emissions associated with a us-based author travelling to a conference are relatively low, the us as a conference hosting country contributes a lot to CO_2_ emissions, both through the number of presented papers and the emissions associated with them. As we can see in Figure 3, the West Coast and Hawaii are leading in these two aspects.

Most CO_2_ emissions in our study are attributed to travel to us-based conferences (Table 9), with travel within the us being the highest source of emissions. Within the us, (Table 10) travel to sunny California is the source of all but one of the top ten locations with the highest CO_2_ emissions; the other is travel *from* California to Florida.

**Table 9:** Most Commonly Travelled Country Pairs and those with the Highest CO_2_ Emissions.

Most Commonly Travelled					Highest CO_2_ Emissions				
Author	Conf.	#	Average	Total	Author	Conf.	#	Average	Total
Country	Country	Samples	CO_2_ kg	CO_2_ t	Country	Country	Samples	CO_2_ kg	CO_2_ t
US	US	10,095	370	3,734	US	US	10,095	370	3,734
JA	US	2,009	1,539	3,092	JA	US	2,009	1,539	3,092
CH	CH	1,371	141	193	GM	US	1,117	1,376	1,537
GM	US	1,117	1,376	1,537	UK	US	745	1,230	916
JA	JA	934	62	58	CH	US	505	1,760	889
UK	US	745	1,230	916	IT	US	572	1,480	847
CA	US	743	458	340	KS	US	475	1,649	783
IT	US	572	1,480	847	FR	US	422	1,339	565
CH	US	505	1,760	889	SN	US	234	2,396	561
KS	US	475	1,649	783	CA	US	743	458	340

**Table 10:** Most Commonly Travelled US State Pairs and those with the Highest CO_2_ Emissions.

Most Commonly Travelled					Highest CO_2_ Emissions				
Author	Conf.	#	Average	Total	Author	Conf.	#	Average	Total
State	State	Samples	CO_2_ kg	CO_2_ kg	State	State	Samples	CO_2_ kg	CO_2_ kg
CA	CA	820	51	41,539	NY	CA	249	656	163,305
NY	CA	249	656	163,305	MI	CA	181	600	108,605
TX	CA	232	417	96,755	TX	CA	232	417	96,755
MI	CA	181	600	108,605	CA	FL	125	654	81,689
AZ	CA	130	165	21,464	MD	CA	120	662	79,386
CA	FL	125	654	81,689	MA	CA	109	693	75,529
MD	CA	120	662	79,386	FL	CA	118	635	74,913
FL	CA	118	635	74,913	VA	CA	102	643	65,565
MA	CA	109	693	75,529	PA	CA	98	654	64,098
TX	TX	106	33	3,508	NC	CA	97	647	62,766

Looking at the most common trips at the country level (Table 9) we find most of the worst offenders in terms of total CO_2_ emissions. However, the list also includes a lot of travel within Switzerland and Japan, which generates an order of magnitude fewer total emissions, and probably even fewer if one takes into account that these trips are often made by train. A similar pattern is not apparent when we look at common trips within the us (Table 10). Travel within California generates a full quarter of the CO_2_ emissions of the worst offender, namely travel from New York to California, indicating the need for improving the state’s rail links.

Finally, we tried to estimate the total carbon footprint of science travel associated with presenting papers at conferences. We calculated the average amount of CO_2_ emissions per conference paper to be 801 kg; this figure comes from data from the 32,264 papers for which we were able to calculate their emissions. To establish the total number of conference papers published in a (recent) year, we undertook an overlap analysis [13] of two bibliographic databases, Scopus and isi Web of Science. We estimated a total of 1.17 million conference papers in 2008 with a 95% confidence interval of 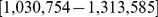.

For this number of conference papers per year the emissions amount to 939 kt CO_2_ in 2008. Total CO_2_ emissions were at 28.962 Gt in 2007, with international aviation emissions totalling 411.6 Mt CO_2_ [14]. Assuming that the increase from 2007 to 2008 followed a 3% annual trend [15], science travel emissions accounted for about 0.003% of all emissions or 0.228% of international aviation emissions in 2008.

This may not seem much. On a per capita basis, however, the total per capita emissions were 4328 kg CO_2_ (2754 kg CO_2_ for non- oecd countries and 10,969 kg CO_2_ for oecd countries) [14]. Since a conference trip corresponds on average to 801 kg CO_2_, the share of conference travel in the mean CO_2_ footprint of an average person is far from negligible. One may counter that scientists are probably a very biased sub-group within the populations of the world, with a higher than average CO_2_ footprint, and therefore the CO_2_ emissions associated with their conference travel form a relatively smaller percentage of their total CO_2_ footprint. However, this argument as an excuse for a scientist’s higher CO_2_ emissions does not hold much water under any of the four prominent proposals for allocating them in the future, namely, equal per capita entitlements, rights to subsistence emissions, priority of the least well-off, or equalizing marginal costs [16].

Science has the duty to understand and explain climate change, to inform policy discussions, and to work out alternatives. This is an important responsibility. Scientists should therefore lead by example in the efforts to solve the problem.

## Materials and Methods

We obtained our primary set of conference paper bibliographic details from the Scopus digital library by retrieving details of conference proceedings papers published over the period 1998–2008. We sampled the papers in a random fashion by selecting those whose author identifier–a system-assigned ten digit integer–last three digits ended in one of the following twenty combinations: 001, 111, 222, …, 999, and 120, 121, …, 129. The sample’s coverage decreases over the years, varying from a high of 5.1% in 1999 to a low of 1.8% in 2008.

We ensured the reproducibility of our Scopus queries by limiting each query’s results to papers entered into the system before July 1st 2009, capturing in effect the state of the database on that particular day. For this we used Scopus’s (undocumented) ORIG-LOAD-DATE predicate, and specified as its argument the date measured in elapsed seconds (1,246,406,400) from January 1st, 1970 (the so-called Unix epoch). Because the results of each query were larger than the number we could download from Scopus, we divided each query into halves, based on the paper’s publication year. Thus a typical query pair would be

pubyear bef 2004 and pubyear aft 1997 and srctype(p) and AU-ID(*120) and ORIG-LOAD-DATE BEF 1246406400.

and

pubyear bef 2009 and pubyear aft 2003 and srctype(p) and AU-ID(*120) and ORIG-LOAD-DATE BEF 1246406400.

To calculate the CO_2_ emissions per conference we assumed that a traveling author requires a single flight to get to the conference venue, and the flight would connect the departure and arrival points of latitude 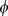 and longitude 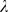 by the shortest possible arc, whose length 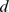 we calculate by using the Haversine formula [17]:
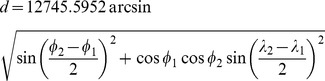



Our assumptions underestimate the actual carbon footprint per travel, as trips seldom use the ideal path, and flight connections add take-offs and landings that increase CO_2_ output. When applying the method described in the *Act on CO*_2_ *Calculator Version 2.0* [18], we distinguished only between short-haul and long-haul flights at 3700 km and assumed that scientists travel only economy class.

To determine the geographical coordinates of the author’s and the conference’s location we used two gazetteers (geographical dictionaries): the us National Geospatial-Intelligence Agency’s (nga) database of foreign geographic feature names and the us Geological Survey (usgs) topical gazetteer files. We also used tables of large us cities from the us Census Bureau, and expanded country and administrative division codes according to the us Federal Information Processing Standard 10–4. In total, out of 63,034 papers in our database, of which 59,522 had data on both the author and the conference location, we fully geolocated 32,264 papers, pinning down 83% of the available conference locations and 61% of the available correspondence addresses. The travel emissions associated with presenting papers at a conference, the corresponding percentage over the total emissions, the average CO_2_ emissions for each paper, and the corresponding number of papers appear in Table 11 in terms of the author’s country and in Table 12 in terms of the conference’s country. Although we include only geolocated papers in our results, the ratio of geolocated papers to the total of our sample for each year is high (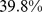 to 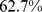, 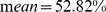, 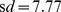; see Table 2 ).

**Table 11:** Conference Travel CO_2_ Emissions by Author Country.

Country	CO_2_ %	Total CO_2_ kg	Average CO_2_ kg	Papers
United States	23.93	6,181,823	510	12,127
Japan	17.69	4,570,831	1,096	4,170
Germany	7.59	1,961,330	983	1,995
China	5.98	1,545,556	668	2,315
United Kingdom	5.37	1,388,650	944	1,471
Italy	4.37	1,129,675	934	1,210
Korea, Republic Of	4.03	1,040,157	1,177	884
Singapore	3.17	819,621	1,669	491
Canada	3.16	817,063	622	1,313
France	2.97	767,766	984	780
Australia	2.08	537,280	1,722	312
Spain	1.98	512,298	862	594
Russia	1.64	424,819	841	505
Switzerland	1.23	317,270	1,102	288
Israel	1.20	311,329	1,483	210
Belgium	1.07	275,760	922	299
India	0.89	228,720	1,197	191
Brazil	0.82	211,810	1,403	151
Netherlands	0.80	207,809	990	210
Portugal	0.79	204,524	838	244
Sweden	0.79	203,352	1,017	200
Taiwan	0.77	198,472	1,369	145
Poland	0.62	160,448	622	258
Finland	0.58	150,601	997	151
Austria	0.57	147,418	910	162
Greece	0.55	141,723	886	160
Turkey	0.49	126,876	1,123	113
Ireland	0.41	106,048	862	123
Chile	0.40	104,348	1,711	61
Norway	0.37	96,127	924	104
New Zealand	0.37	95,903	1,880	51
Egypt	0.25	65,089	1,228	53
Czech Republic	0.25	64,800	689	94
Thailand	0.25	64,773	1,580	41
Hungary	0.23	59,434	668	89
South Africa	0.22	56,718	1,891	30
Argentina	0.20	50,670	1,535	33
Ukraine	0.19	48,012	717	67
Slovenia	0.17	43,539	764	57
Malaysia	0.16	40,615	967	42
Denmark	0.14	36,961	924	40
Romania	0.13	34,485	515	67
Bulgaria	0.09	23,451	838	28
Belarus	0.07	17,748	592	30
Mexico	0.06	15,754	716	22
Estonia	0.05	13,419	479	28

**Table 12:** Conference Travel CO_2_ Emissions by Conference Country.

Country	CO_2_ %	Total CO_2_ kg	Average CO_2_ kg	Papers
United States	65.52	16,928,118	875	19,350
Canada	4.61	1,190,041	850	1,400
Australia	3.39	876,685	1,902	461
Japan	3.08	795,323	526	1,513
China	2.92	753,170	391	1,925
France	2.16	557,336	715	780
United Kingdom	1.97	509,788	696	732
Germany	1.59	410,558	570	720
Italy	1.47	380,322	627	607
Korea, Republic Of	1.37	353,958	640	553
Spain	1.33	344,304	679	507
Netherlands	0.76	197,283	725	272
Mexico	0.68	174,905	1,041	168
Turkey	0.63	163,270	912	179
Singapore	0.60	155,950	830	188
Sweden	0.60	153,804	684	225
Portugal	0.54	139,145	632	220
Taiwan	0.54	138,376	1,081	128
Austria	0.52	133,960	736	182
Belgium	0.49	126,647	728	174
India	0.43	111,307	876	127
Argentina	0.43	111,296	1,795	62
Brazil	0.42	108,068	1,403	77
Switzerland	0.39	101,635	907	112
Thailand	0.38	98,957	1,137	87
Greece	0.27	70,201	798	88
Poland	0.27	68,492	349	196
Russia	0.26	68,133	344	198
Finland	0.25	65,519	736	89
Czech Republic	0.21	53,366	550	97
Norway	0.20	52,746	713	74
Hungary	0.18	45,519	511	89
Ireland	0.15	38,315	518	74
Romania	0.13	33,093	480	69
Malaysia	0.13	33,092	827	40
Denmark	0.13	32,951	646	51
Cyprus	0.10	25,688	803	32
Belarus	0.09	23,165	579	40
Slovenia	0.09	22,703	445	51
Croatia	0.04	10,582	278	38
Ukraine	0.03	8,435	312	27
Serbia	0.02	5,221	180	29

We matched conference locations in the gazetteers among many locations with the same name using a series of increasingly rough heuristics looking for: a unique name and state (e.g. Anaheim, ca), a unique name (e.g. Kuala Lumpur), a country’s capital (Paris), or for a unique or major city and a country (Beijing, China). Author addresses in our data set were always tagged with a country, and we therefore matched them looking either for a city in a specified state and country, or for a unique or major city and a country (Beijing, China). In addition, we cleaned up postcodes located adjacent to city names by matching them according to country or region–specific standards, and we created various aliases for countries and administrative regions, which would cause violent convulsions to many diplomats.

To establish the total number of conference papers published in a (recent) year, we undertook an overlap analysis of two bibliographic databases, Scopus and isi Web of Science. We proceeded as follows.

If 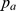 is the fraction of all papers in the world indexed by the first database and 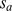 is the number of papers in the first database (its size), then the total number of papers in the world is 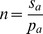. Assuming that each database indexes independently, then if 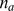 is the number of papers returned for a query by the first database, 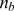 is the number of papers returned for the same query by the second database, and 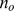 is the number of papers returned for the same query by both databases, we have 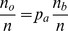 so that 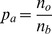. Substituting we get 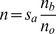.

We executed queries in both Scopus and isi Web of Science in February and March of 2010. Since both databases limit the number of results that can be downloaded for each query, we took into account only queries returning no more than 500 papers. We also took out of the calculations queries returning less than 50 papers, as in this case the overlap (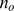) could be very small creating outliers. Papers were matched if they were published in the same year and they had the same start and end page.

The queries were single words that we required to be matched exactly, for material published in proceedings in 2008. The words were selected by trawling the titles of paper titles that were published in 2008 in the journals Science and Nature. In Scopus, the queries were of the form:

TITLE({science}) AND SRCTYPE(p) AND PUBYEAR IS 2008

while in ISI the queries were of the form:

TI = science AND PY = 2008

having selected the Conference Proceedings Citation Index–Science (cpci-s)–1990–present and Conference Proceedings Citation Index–Social & Humanities (cpci–ssh)–1990–present.

In the end, we had 80 result sets that met our criteria. From these we estimated a total of 1,172,169 conference papers in 2008 with a 95% confidence interval of 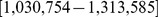.
